# Room-temperature bonding of Al_2_O_3_ thin films deposited using atomic layer deposition

**DOI:** 10.1038/s41598-023-30376-7

**Published:** 2023-03-03

**Authors:** Ryo Takakura, Seigo Murakami, Kaname Watanabe, Ryo Takigawa

**Affiliations:** Graduate School of Information Science and Electrical Engineering, Kyushu University, 744 Motooka, Nishi-ku, Fukuoka 819-0395 Japan

**Keywords:** Electronic devices, Electrical and electronic engineering

## Abstract

In this study, room-temperature wafer bonding of Al_2_O_3_ thin films on Si thermal oxide wafers, which were deposited using atomic layer deposition (ALD), was realized using the surface-activated bonding (SAB) method. Transmission electron microscopy (TEM) observations indicated that these room-temperature-bonded Al_2_O_3_ thin films appeared to work well as nanoadhesives that formed strong bond between thermally oxidized Si films. The perfect dicing of the bonded wafer into dimensions of 0.5 mm × 0.5 mm was successful, and the surface energy, which is indicative of the bond strength, was estimated to be approximately 1.5 J/m^2^. These results indicate that strong bonds can be formed, which may be sufficient for device applications. In addition, the applicability of different Al_2_O_3_ microstructures in the SAB method was investigated, and the effectiveness of applying ALD Al_2_O_3_ was experimentally verified. This successful SAB of Al_2_O_3_ thin films, which is a promising insulator material, opens the possibility of future room-temperature heterogenous integration and wafer-level packaging.

## Introduction

Room-temperature bonding technology has emerged as a challenging process for three-dimensional integration and wafer-level packaging of optoelectronics, MEMS, RF, and CMOS devices. As a technique for direct bonding, hydrophilic bonding via plasma activation is becoming mainstream in the semiconductor industry^1–3^. This method requires annealing at a temperature of a few hundred degrees to achieve a sufficiently strong bond for practical applications. To overcome the severe thermal stress and degradation of bond alignment accuracy, the development of room-temperature bonding technology is essential.

As one solution, surface-activated bonding (SAB)^4^ is a promising room-temperature direct bonding technology. In this method, wafer surfaces are first irradiated using an argon (Ar) fast atom beam (FAB) to remove organic contaminants and the native oxide layer, and then, the activated wafer surfaces are brought into contact at room temperature. The applicability of this method to various materials has been studied for a wide range of device applications. To date, the SAB method has demonstrated room-temperature bonding of metals (Cu–Cu^5,6^ and Au–Au^7,8^) as well as semiconductors (Si–Si^9^, Ge–Ge^10^, Si–GaAs^11^, Si–SiC^12^, and GaAs–SiC^13^). However, it is difficult to apply this method to the direct bonding of SiO_2_ and SiN, which are suitable as insulator layer materials in semiconductor device applications^14,15^. To overcome this limitation, modified SAB^16–19^ and atomic diffusion bonding^20,21^ have been reported based on the quasi-direct bonding concept using a metal intermediate layer. In addition, a room-temperature bonding method using an Si intermediate layer has recently been reported^22–25^. In some electronics applications such as 3D integration using vertical electrical interconnections, these intermediate layers cause serious current leakage. Therefore, the SAB of insulating materials is highly desirable.

Al_2_O_3_ is a promising alternative insulator material because of its excellent electrical resistivity and thermal conductivity, which are comparable to those of SiO_2_. To date, the SAB of single-crystal Al_2_O_3_^26^, SAB of Al_2_O_3_ deposited by mist CVD^27^, and hydrophilic bonding of Al_2_O_3_^28^, which requires annealing at 100–300 °C after temporary bonding, have recently been examined. Herein, we focus on Al_2_O_3_ thin films grown by atomic layer deposition (ALD), which allows for precise growth, atomic-scale thickness control, and good uniformity on large-scale wafers. Previous reports on Al_2_O_3_ thin films deposited by ALD, have investigated hydrophilic bonding^29,30^ and modified SAB^31^, in which an adhesive of Si is sputter-deposited on ALD-deposited Al_2_O_3_ films. SAB^32^ of AlO films deposited by ion beam sputtering has also been examined. It is interesting to apply ALD Al_2_O_3_ thin films to the SAB method.

There have only been a few studies on the application of the SAB method to the direct bonding of ALD Al_2_O_3_ thin films without a heating process, and a large bond strength has not yet been achieved. Herein, the applicability of the SAB method to the direct bonding of ALD Al_2_O_3_ thin films is investigated in comparison with those of single-crystal sapphire. Furthermore, we demonstrate the room-temperature wafer direct bonding of deposited Al_2_O_3_ thin films on a Si thermal oxide wafer using the ALD method. Compared to previous reports, the novelty of this study is the utilization of a surface-activated bonding method using Ar-FAB for Al_2_O_3_ thin films deposited by ALD.

## Results and discussion

**Figure 1:**
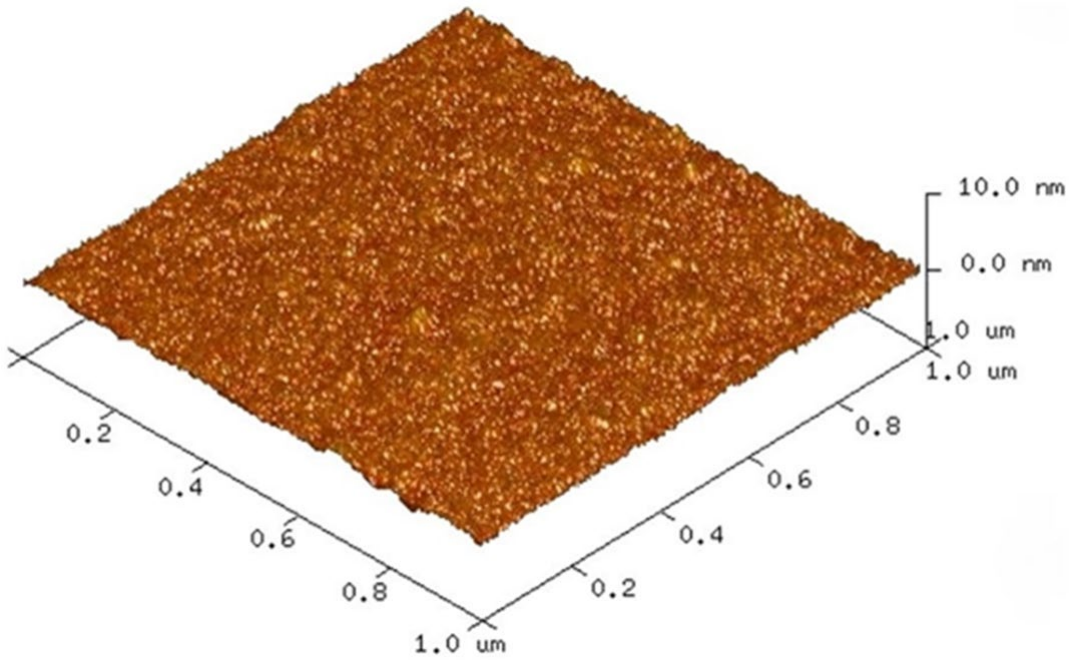
AFM image of the deposited Al_2_O_3_ thin film using ALD method.

SAB of ALD Al_2_O_3_ thin films on 4-inch Si thermal oxide wafers was performed at room temperature. Ar FAB irradiation was used for surface activation. Surface smoothness is one of critical factors for room-temperature direct bonding, and a rms of approximately 0.5 nm or less is typically required^33^. First, the wafer surfaces were studied before and after Ar FAB irradiation using atomic force microscopy (AFM). Figure 1 shows an AFM image of the ALD Al_2_O_3_ surface over a measured area of 1 µm × 1 µm. Consequently, the RMS surface roughness of the ALD Al_2_O_3_ thin film was 0.32 nm and 0.25 nm before and after the Ar FAB irradiation, respectively. This indicates that Ar FAB irradiation for surface activation did not roughen the surface significantly, and the surface of the ALD Al_2_O_3_ film retained sufficient smoothness.

**Figure 2:**
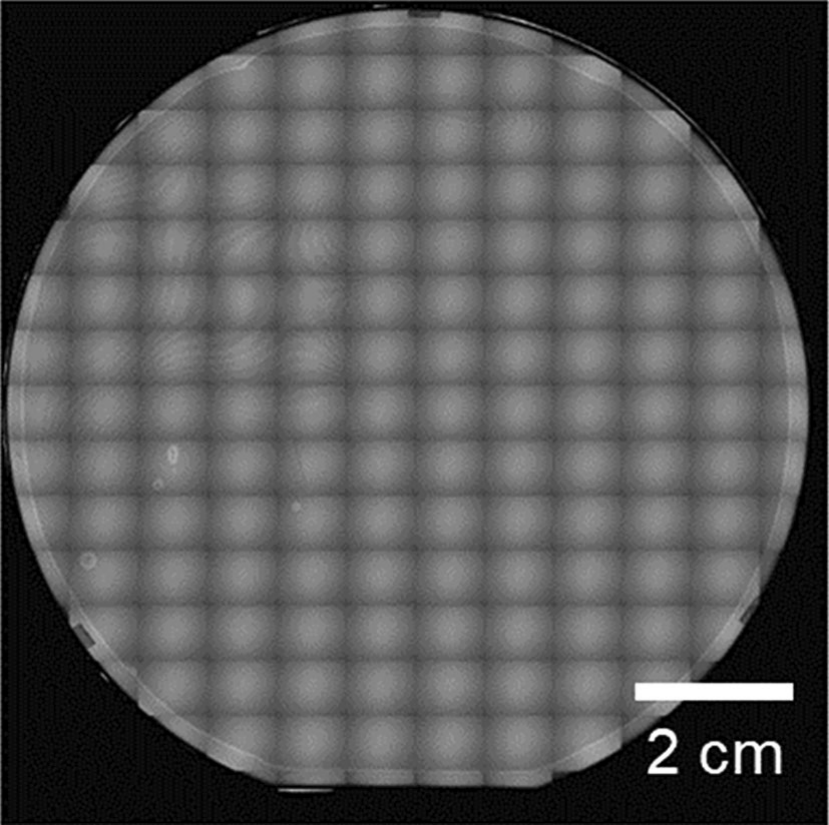
IR image of 4-inch-scale bonded thermal oxidized Si–Si wafer with ALD Al_2_O_3_ thin films at room temperature.

**Figure 3:**
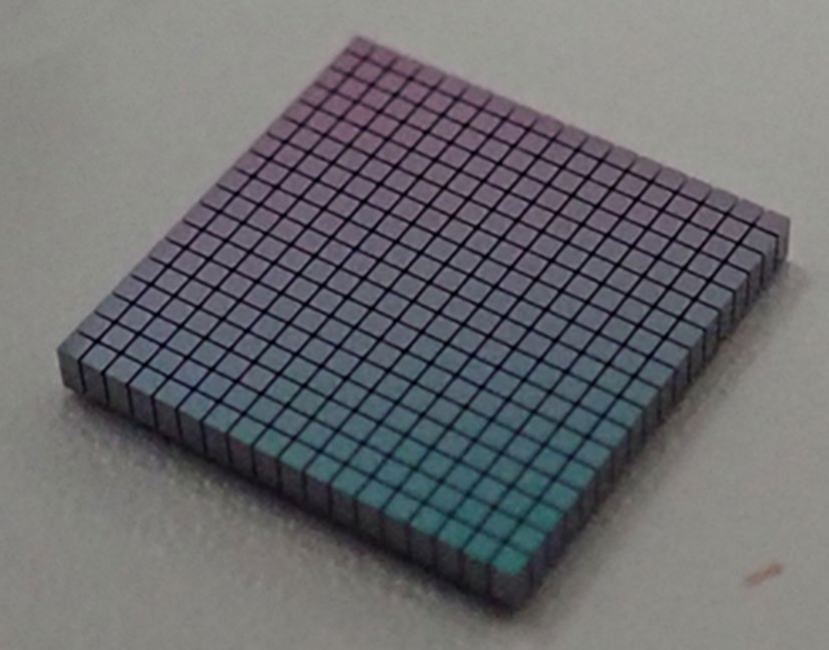
Image of the diced 0.5 mm × 0.5 mm chips, showing perfect dicing without debonding.

Figure 2 shows the infrared (IR) transmission image of the SiO_2_–SiO_2_ interface bonded with ALD Al_2_O_3_ thin films, showing no large voids in the entire bonded wafer. The bond strength was evaluated using the crack-opening method^34^ and the half-cutting dicing test. In the crack-opening method, a razor blade is inserted into the bond interface, and the surface energy, which indicates the bond strength, can be calculated using the propagated crack length. Using the measured crack length, the estimated surface energy of ALD Al_2_O_3_–ALD Al_2_O_3_ was approximately 1.5 J/m^2^. The dicing test of the bonded wafer was then performed using a dicing saw. The bonded wafer was cut into 10-mm-square chips, and then one chip was half-cut into 0.5-mm-square pieces, leaving the bottom of the chip. The durability against the applied stress during the dicing process indicates the bond strength. As shown in Fig. 3, perfect dicing was successful with no debonding or chipping observed. These results indicate that a strong bond between ALD Al_2_O_3_ thin films can be achieved, which may be sufficient for device applications.

**Figure 4:**
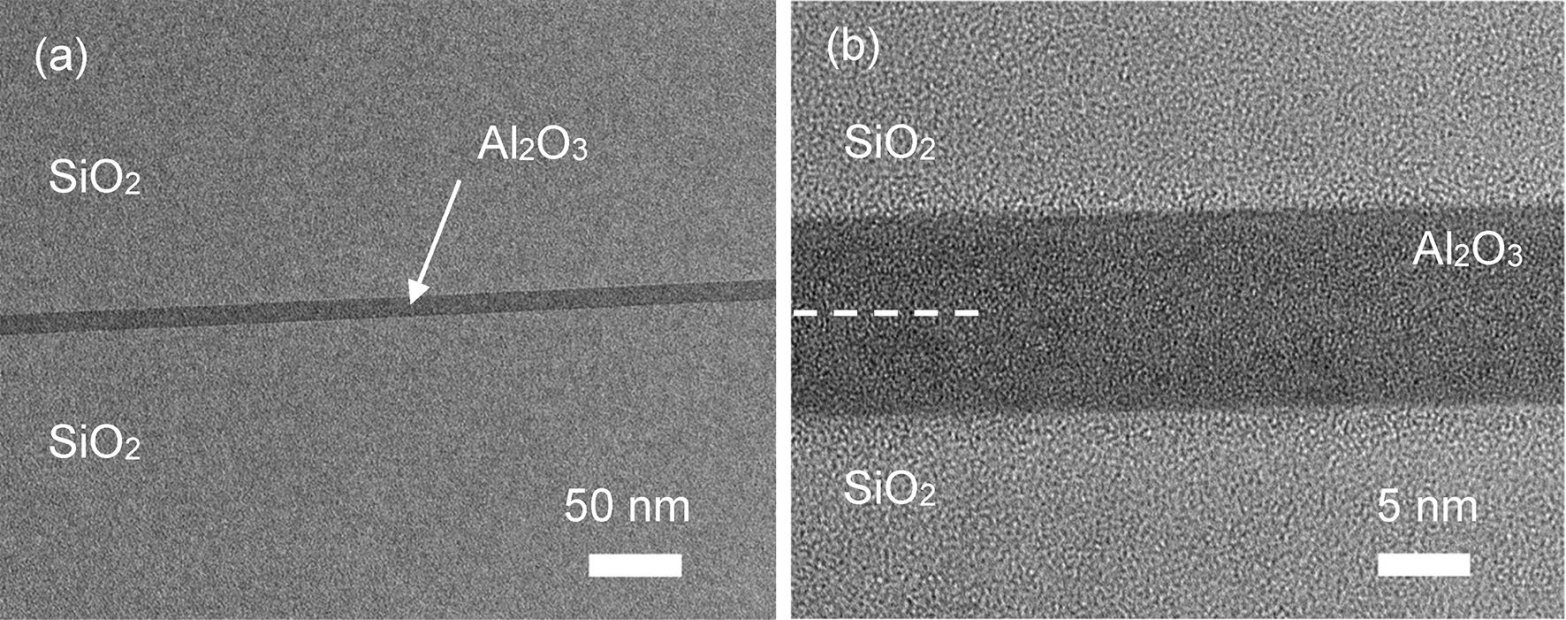
(**a**) Low- and (**b**) high-magnification cross-sectional TEM images of the bonding interface.

The nanostructure of the cross-sectional bonding interface was observed using transmission electron microscopy (TEM). Figure 4a,b show the TEM images obtained at low and high magnifications, respectively. The dotted line in Fig. 4b shows the initial bond interface of the ALD Al_2_O_3_ thin film.

As shown in Fig. 4b, a void-free bonding interface could be achieved at the atomic level, indicating the presence of a strong bond. In addition, we can see no damaged layer by Ar FAB irradiation, whereas this damaged layer clearly exists in the SAB of sapphire–sapphire.

**Figure 5:**
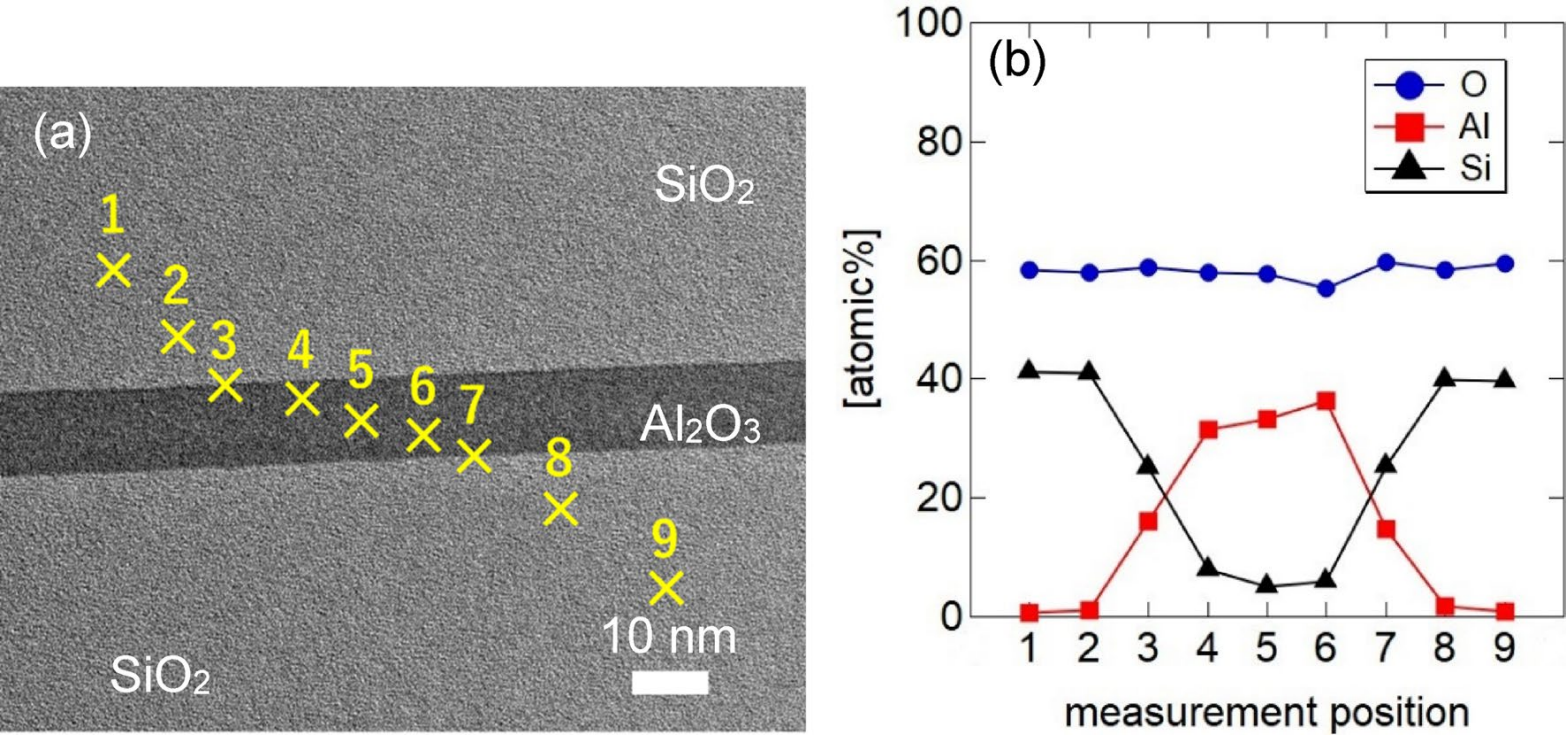
Elemental analysis using cross-sectional STEM-EDX.

These results indicate that the bonded ALD Al_2_O_3_ thin films functioned as adhesives and facilitated strong bond formation between two SiO_2_ films. Figure 5 shows the results of elemental analysis obtained using energy-dispersive X-ray spectroscopy (EDX). Figure 5a shows a scanning TEM (STEM) image; the numbers in the image indicate EDX measurement points, and Fig. 5b shows the element concentrations at each measurement point. The elemental analysis of the bonded ALD Al_2_O_3_ in the depth direction (Fig. 5b) shows that there is little change in composition from the bonding interface in the depth direction (measurement points 4–6).

**Figure 6:**
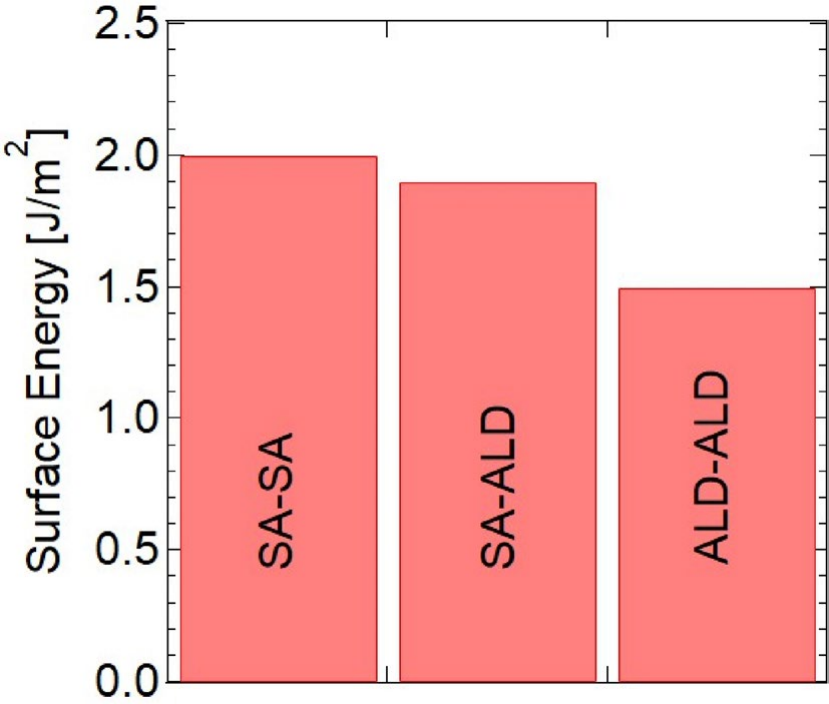
Comparison of the bond strengths of different Al_2_O_3_ samples.

**Figure 7:**
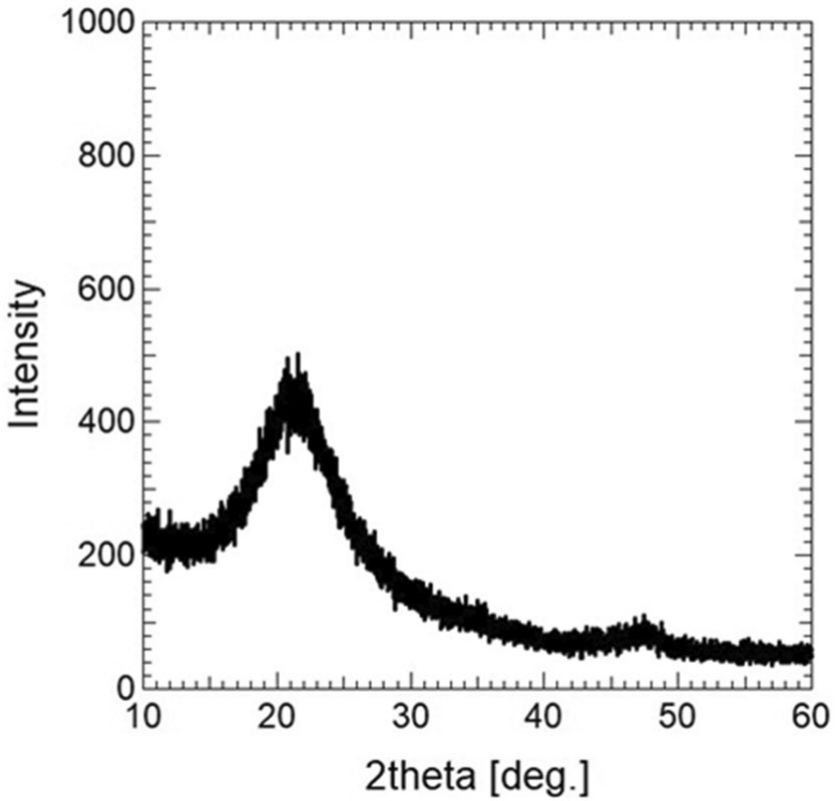
XRD pattern of ALD Al_2_O_3_ thin film.

The bond strengths of the different Al_2_O_3_ were investigated. Figure 6 compares the bond strengths of sapphire–sapphire (SA–SA), sapphire–ALD Al_2_O_3_ (SA–ALD), and ALD Al_2_O_3_–ALD Al_2_O_3_ (ALD–ALD). Using the crack-opening method, the estimated surface energies of the bonded SA–SA, SA–ALD, and ALD–ALD were approximately 2.0, 1.9, and 1.5 J/m^2^, respectively. It is well-known that the typical Al_2_O_3_ thin film deposited by ALD is amorphous. As shown in Fig. 4, no lattice structure was visible in the bonded ALD Al_2_O_3_ thin film, indicating that it was amorphous. As shown in Fig. 7, the measured X-ray diffraction (XRD) results also indicated that the Al_2_O_3_ thin film deposited by ALD is amorphous.

On the other hands, although sapphire has a single-crystalline structure, an amorphous-like layer (thickness ≈ 1 nm) exists at the bond interface owing to Ar FAB irradiation^27^. We can see that this crystal defect layer has little effect on the bonding strength because sufficient bond strength can be achieved compared with that of amorphous Al_2_O_3_ thin films. We confirmed that the Al_2_O_3_ thin film-sapphire bond was weaker than the sapphire–sapphire bond. These results suggest that the crystallinity of the activated surface affects the bond strength of Al_2_O_3_. In addition, the experimental results demonstrated that the ALD method is suitable for the SAB of Al_2_O_3_ thin films.

**Figure 8:**
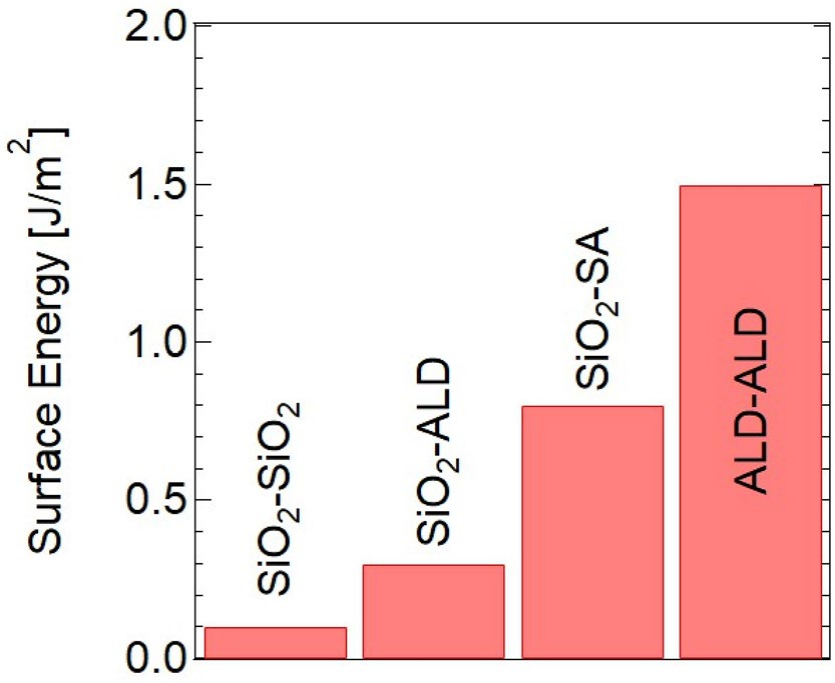
Comparison of bond strengths between SiO_2_ and different Al_2_O_3_ samples.

Next, SAB of SiO_2_–SiO_2_, SiO_2_–ALD Al_2_O_3_ (SiO_2_–ALD), SiO_2_–SA (SiO_2_–sapphire), and ALD Al_2_O_3_–ALD Al_2_O_3_ (ALD–ALD) was performed under the same bonding conditions. Figure 8 shows a comparison of the bond strengths between SiO_2_ and different Al_2_O_3_ samples. The low bond strengths of SiO_2_–SiO_2_ and SiO_2_–ALD were confirmed, with the bond strength of SiO_2_–ALD being slightly larger. Comparatively, the bond strength of SiO_2_–SA was larger than both. This indicates that simply changing one of the wafers to one with better crystallinity tends to increase bond strength. However, it was necessary to form ALD Al_2_O_3_ films on both SiO_2_ sides and bond them to achieve a sufficient strong SiO_2_–SiO_2_ bond interface for device applications. These results also suggest that the single-crystalline phase of Al_2_O_3_ is more effective, and crystallinity is important from the viewpoint of bond strength.

## Conclusion

Herein, the SAB method was successfully applied to achieve the room-temperature wafer bonding of ALD Al_2_O_3_ thin films. The bonded ALD Al_2_O_3_ thin films appear to work well as nanoadhesives and form a strong bond between SiO_2_ surfaces. The bonded wafer was perfectly diced into dimensions of 0.5 mm × 0.5 mm, and the surface energy, indicative of the bond strength, was estimated to be approximately 1.5 J/m^2^. These results indicate that strong bonds that are sufficient for device applications can be achieved. In addition, the applicability of different Al_2_O_3_ microstructures to the SAB method was investigated, and the effectiveness of applying ALD Al_2_O_3_ was experimentally verified. This successful SAB of Al_2_O_3_ thin film, which is a promising insulator material, opens the possibility of future room-temperature heterogenous integration and wafer-level packaging. In additions, the results of this work will be of significant use in the development of fabrication technique for X materials-on-insulators using room temperature bonding method with ALD Al_2_O_3_ thin film, and not only SiO_2_–SiO_2_ bond interface.

## Methods

In our experiments, we used an ultrathin Al_2_O_3_ film (thickness = 6 nm) deposited by atomic layer deposition (ALD) on a 4-inch wafer with a 1-µm thermal oxide film. The ALD deposition process used trimethylaluminum (TMA) as a precursor and ozone (O_3_) as an oxidant. The deposition temperature was 400 °C. Post-deposition annealing was not performed.

Bonding was performed using a wafer-level bonding apparatus (MWB-08-AX, NIDEC MACHINE TOOL CORPORATION). For the bonding process, two wafer surfaces were simultaneously irradiated with Ar-FAB in a vacuum chamber at 5.1 × 10^−6^ Pa. The FAB irradiation conditions were as follows: voltage was 1.5 kV, current was 100 mA, Ar flow rate was 13 sccm, and FAB irradiation time was 30 s.. After FAB irradiation, the FAB-irradiated surfaces of the wafers were immediately brought into contact with each other in a vacuum apparatus, and a load of 9.8 × 10^4^ N was applied for 10 s. for bonding.

X-ray diffraction (XRD) method was used to evaluate the crystallinity of ALD Al_2_O_3_ thin films. In-plane measurements were performed with an X-ray incidence angle of 0.5 degrees (the critical angle was 0.4 degrees).

The roughness of the deposited surface was evaluated using atomic force microscopy (AFM, DimensionIcon, Bruker). The nanostructure and elemental composition of the bonding interface were analyzed by TEM (Hitachi Hitech H9500) and STEM-EDX (Hitachi Hitech G4000).

## Data Availability

The datasets used and/or analyzed during the current study available from the corresponding author on reasonable request.
